# Implementing a social network intervention: can the context for its workability be created? A quasi-ethnographic study

**DOI:** 10.1186/s43058-020-00087-5

**Published:** 2020-10-27

**Authors:** J. Ellis, I. Vassilev, E. James, A. Rogers

**Affiliations:** NIHR CLAHRC Wessex, School of Health Sciences, University of Southampton, University Road, Building 67, Southampton, SO17 1BJ UK

**Keywords:** Community setting, Social networks, Commissioning, Consolidated Framework for Implementation Research

## Abstract

**Background:**

Policy makers and researchers recognise the challenges of implementing evidence-based interventions into routine practice. The process of implementation is particularly complex in local community environments. In such settings, the dynamic nature of the wider contextual factors needs to be considered in addition to capturing interactions between the type of intervention and the site of implementation throughout the process. This study sought to examine how networks and network formation influence the implementation of a self-management support intervention in a community setting.

**Methods:**

An ethnographically informed approach was taken. Data collection involved obtaining and analysing documents relevant to implementation (i.e. business plan and health reports), observations of meetings and engagement events over a 28-month period and 1:1 interviews with implementation-network members. Data analysis utilised the adaptive theory approach and drew upon the Consolidated Framework for Implementation Research. The paper presents the implementation events in chronological order to illustrate the evolution of the implementation process.

**Results:**

The implementation-network was configured from the provider-network and commissioning-network. The configuration of the implementation-network was influenced by both the alignment between the political landscape and the intervention, and also the intervention having a robust evidence base. At the outset of implementation, the network achieved stability as members were agreed on roles and responsibilities. The stability of the implementation-network was threatened as progress slowed. However, with a period of reflection and evaluation, and with a flexible and resilient network, implementation was able to progress.

**Conclusions:**

Resilience and creativity of all involved in the implementation in community settings is required to engage with a process which is complex, dynamic, and fraught with obstacles. An implementation-network is required to be resilient and flexible in order to adapt to the dynamic nature of community contexts. Of particular importance is understanding the demands of the various network elements, and there is a requirement to pause for “reflection and evaluation” in order to modify the implementation process as a result of learning.

Contributions to literature

- Implementation literature pays attention to the role of context in the implementation process, and this paper demonstrates how context is created by the networks involved.
- Implementation frameworks exist and are useful for guiding the process in established settings. This paper goes further by illustrating the process of making ready a setting when the environmental conditions required do not exist.
- The role of intervention pliability and context adaptability is well documented, and this paper pays close attention to the relationship between network elements and the formation and how these influence the need for adaptability.

## Background

There is recognition among policy makers and researchers that the process of implementing and sustaining evidence-based interventions into practice faces many challenges due to the interactions between intervention type, implementation site, and the local and wider context [1–3]. In particular, the context has been described as critical for implementation, with a recognition that interventions have the potential to be shaped and transformed by the environment into which they are introduced [4]. As such, the nature of contexts has been a focus for frameworks designed to guide and understand how interventions are implemented [5, 6]. The current study captured the process of implementing a social network intervention aimed at promoting self-management support (SMS) in a community setting.

Understanding the implementation in community settings requires further exploration for several reasons. First, as an open system, that is more entangled and messier than a closed-system where entities and processes are independent from one another [7], there are non-linear and dynamic relationships between system elements [8], including challenges associated with working across organisational boundaries [9–11]. It is recognised that community implementation is therefore fluid, and the delivery requires flexibility from both the intervention and the context to fit the needs of local populations [12]. As Ham and Murray [13] point out, there is no ‘blue print’ for implementation, and the current policy emphasis is placed on diverse solutions and local leadership’, supported by ‘meaningful local flexibility’ in the way in which implementation is to occur [12]. While this allows for tailoring implementation to local needs, it could also place insufficient attention on ensuring the necessary strategies for maximising the adoption, embedding and successful implementation of initiatives. This absence of a blueprint could also potentially make implementation in community settings more unpredictable than closed settings such as hospitals.

Second, community-oriented SMS involves contact with the voluntary and community sector and encourages individuals to connect to, and engage with, their local community. Consequently, the role of network processes are likely to be drivers of implementation [14] and therefore crucial to explore, for example; understanding the way different organisations interact as a means of improving communication, building and reshaping existing relationships and increasing knowledge regarding the developing value of the intervention and sense of co-ownership [15, 16]. With a focus on ‘local flexibility’, it is relevant to understand how SMS interventions can be successfully implemented in the light of the acknowledgement that these are likely to have (unfulfilled) potential to address the physical, social and psychological needs of people with long-term conditions [17, 18]. Added to this is the acknowledgement that implementation is taking place at a time when there is increased pressures on, and reduced capacity of, commissioners, providers and users of services to work collaboratively and creatively to develop and implement services [19]. This study sought to illuminate the emergent network processes, organisational arrangements and strategies implicated in attempts to implement a SMS intervention within a local health and social care system.

## Methods

### Aim

The study aimed to examine how networks and network formation influence the implementation process.

### Study setting

This case-study followed the attempt to implement a SMS intervention in a commissioning community in the South of England, the purpose of which was to learn about the process in order to inform future implementation efforts of this and similar interventions. In England, the 2012 Health and Social Care Act [20] established Clinical Commissioning Groups (CCGs), clinically led statutory bodies, in order that decision making was localised. This system has been described as a quasi-market due to the separation between commissioners (purchasers) and providers of service [21] (quasi because commissioners are meant to act on behalf of patients). Since 2012, there have been opportunities for a range of public, private, and charity providers to compete to provide NHS services [21]. This competition is managed by the CCGs who have a strategic role in assessing local population needs, planning appropriate services and contracting providers. The Better Care Fund [22] introduced a new partnership where budgets were pooled and the CCG and local authorities were required to work together to commission health and social care services for the local population. This new partnership is the ‘commissioning-network’ referred to here.

### Study participants

The study follows two networks, the commissioning-network and the provider-network, who came together to create a new implementation-network that is focus on this study.

The commissioning-network was made up of elected local councillors, commissioners from the CCG (holding different degrees of responsibility and specialism) as well as service providers from local community groups and organisations. In consideration of anonymity, the details of the commissioning-network remain purposively broad.

The provider-network was based at the National Institute for Health Research (NIHR) Collaboration for Leadership in Applied Health Research and Care Wessex (CLAHRC) and consisted of the academics who developed the intervention, a business manager and a project manager whose roles were to support the implementation process. The NIHR’s CLARHC programme ran over a 5-year period and focused on research and implementation to improve health and the quality and cost-effectiveness of health care. With a focus on implementation, the provider-network sought to make intervention a sustainable non-for-profit social enterprise beyond the life of the CLARHC programme.

The study focuses on the activity and influences of these two networks.

### Implementation strategy

The intervention in this study is an online tool (Genie—Generating Engagement in Network Involvement) that maps social networks, allows the user to select their preferences and needs and helps that person engage with local support resources. (See Table 1 for more detail on the intervention). The original strategy adopted by the implementation network was determined solely by the commissioning-network. The provider-network supported the commissioning-network to determine the implementation strategy by making available a set of educational resources explaining the function of the social network approach, the roles required for implementation and its use, and how to best deliver the intervention. The provider network also made available training to demonstrate Genie and upskill individuals in the facilitation process. Guidance detailing the possible stages of implementation was given to the commissioning team as a resource (available as additional material).

**Table 1:** Reporting of the intervention using TIDieR

TIDieR item	Intervention details
1. Name	Generating Engagement in Network Involvement (Genie)
2. Why	Genie has been developed on the evidence that health and wellness is affected by social network properties and types [17, 18]
3. What (materials)	Web-based tool available at: www.genie.soton.ac.uk
4. What (procedure)	Three-staged process: (1) user maps their existing network on a set of concentric circles, (2) answer a 13-item preference questionnaire to ascertain personal interests and (3) based on the answers to this questionnaire opportunities for social engagement in their local area are presented on a google style map.
5. Who provided	Facilitated and co-produced with the participant.
6. How: models of delivery	Delivered and facilitated in perso
7. Where: type of location	In the community (i.e. user’s homes and community venues)
8. When and how	A one-off facilitation process taking approximately 45 min.

The original implementation strategies are as follows:

1. Tender and recruit for a new role, ‘community navigators’, that would be fulfilled by six new people.
2. The community navigators would work directly in the community and would be responsible for connecting people in need to relevant supportive services.
3. People would be referred to the community navigators from primary care and providers of social care.
4. The community navigators take responsibility for the intervention and would:
  - Identify services and support groups whose users could benefit from Genie
  - Train individuals in those services to become Genie facilitators

For the commissioning-network Genie was one element of a personalised care plan provision and one tool to be used alongside others. Thus, the commissioning network purposively left the intervention arrangements (roles and pathways) flexible in order to accommodate the emerging configuration of staff roles following the tender. How this implementation strategy played out was the focus of this study.

### Study design

In implementation science, quasi-ethnographic methods have been used to illuminate the complexity of economic, social and material contexts [23], and intervention flexibility and fidelity in context [24]. Further to its ability to help define complex problems and investigate factors contributing to the problem [25], the tradition advocates the use of multiple methods to develop thick descriptions. The approach taken here is described as quasi-ethnographic because it adopts key principles of building thick descriptions over time, but it deviates from traditional ethnography in regard to the time and intensity of the data collection. The approach affords the opportunity to study the process in context, illuminating the social processes, influences and relationships between entities that impact upon implementation. Three data collection techniques were employed, and JE collected data between March 2017 and May 2019.

Initially, data collection involved obtaining and analysing documents relevant to the implementation. These included Genie’s standard operating procedure (SOP) and business plan to illuminate the intentions of the intervention team, as well as four local health and wellbeing reports to give a contextual understanding of the local population, and the strategic health service plan produced by the commissioning network to provide an account of their intentions.

Subsequent to this participant observation was conducted over the 28-month period, which contributed towards the bulk of the data collection. In line with the dual position adopted by other researchers [26], JE was both being a member of the Genie-network *and* a researcher studying the implementation process. The insider position allowed JE to witness the intricacies of the intervention-provider network to help produce thick descriptions otherwise not possible to obtain as an outsider. JE joined and observed meetings between the provider network and commissioning network to observe and take notes on details pertaining to how the implementation was negotiated, what were important considerations and the relationships between individuals. Observations during provider network’s meetings were also made and notes taken on matters concerning how the team responded to the commissioning team’s requirements and the implementation process. All notes were handwritten in situ.

The third method was 1:1 semi-structured interviews that were carried out with commissioners, local authority councillors and members of the provider network. The first interviews were conducted as initial contract negotiations were underway. Interviews were conducted with the Genie project manager, lead commissioner and appointed councillor to illuminate perceptions around the intervention, implementation intentions and key considerations the individual held regarding the process. The interview schedule was developed in collaboration with the academic members of the provider-network (additional material). Interviews were conducted at the mid-way point with the commissioner to reflect on the process as it was evolving and to follow upon observational points of interest, as well as with a councillor to understand the political landscape. The schedules used here were broad to allow for the exploration of matters arising by happenstance. Interviews were conducted in person or over the phone, lasted between 60 and 90 min and were audio-recorded and transcribed verbatim. Notes were taken directly after to capture non-verbal elements.

The University of Southampton’s Research and Governance Office granted the study ethical approval. Verbal agreement for observations was given at the start of each data collection point by those present. Written consent was sought at the start of interviews, where the participants agreed for data to be used in publications. In order to promote anonymity data excerpts are attributed to a team rather than an individual.

### Data analysis

Data analysis constituted an iterative process utilizing an adaptive theory approach [27]. Following a continual process of familiarization and coding (inductively and deductively), data analysis informed the onward data collection. Deductive coding drew upon the Consolidated Framework for Implementation Research (CFIR) [5]. The CFIR has five domains (Table 2) and recognizes implementation as a social process that is entangled with the context in which it takes place. The CFIR thus was a suitable framework to illuminate the interacting influences on the implementation process [5]. Following a process of inductive coding, the pragmatic application of network theory was applied to interpret the findings. A network is defined as a group of members and the relationship between them. Networks are constantly evolving. They can vary in size, interest and focus as well as stability. A key focus of network theories is to understand how networks form, the associations and relationships between network components and to understand how networks are (re)configured over time. Network theories call for the gaze of implementation research to be focused on events, and the unit of analysis being the network that creates the events [28]. Here, the analysis drew upon network theory to frame the CFIR concepts and view them as network elements. Therefore, the pragmatic application of network theory in combination with the CFIR enabled an understanding of the relationship between network elements to be built, and also an understanding on how the network, and all the influential elements, created the events experienced in the implementation process. This in turn enabled an insight into the implementation process, and how and why Genie was or was not implemented.

**Table 2:** CFIR constructs

CFIR domain	Sub-concept
Intervention characteristics	Intervention source Evidence strength and quality Relative advantage Adaptability Trialability Complexity Design quality and packaging Cost
Outer setting	Patient needs and resources Cosmopolitanism Peer pressure External policies and incentives
Inner setting	Structural characteristics Networks and communications Culture Implementation climate (relative priority, organisational incentives and rewards, goals and feedback, learning climate) Readiness for implementation (leadership engagement, available resources, accessible information and knowledge)
Individual characteristics	Knowledge and beliefs about the intervention Self-efficacy Individual stage of change Individual identification with organisation Other personal attributes
Process of implementation	Planning Engaging (opinion leaders, formally appointed internal implementation leaders, champions, external change agents) Executing Reflecting and evaluating

Data analysis was conducted by JE and was shared and discussed regularly with authors to sense check and develop. The qualitative software NVIVO.11 was used to assist in data organization.

## Results

The results outline how the provider network and the commissioning network configured to attempt to implement the intervention and in doing so co-created the events experienced during the implementation process. The results are structured according to the four events distinguished and presented in chronological order [14]. The first event discusses the configuration of the implementation network, the second outlines how the implementation-network achieved stability and the third and fourth events discuss threats to this stability and network resilience. Table 3 offers a summary of the events and the relevant CFIR concepts.

**Table 3:** Implementation events and influencing CFIR concepts

Implementation event	Key points	CFIR concept
Event 1	Configuration of implementation network	External policies (+) Evidence strength and quality (+)
Event 2	The implementation network achieving network stability	Implementation climate (+) Resource availability (+)
Event 3	Network resilience required to overcome barriers to implementation	Reflecting and evaluating (+) Readiness for implementation (−)
Event 4	Reconfiguration of implementation network and division of labour to increase network stability and progress implementation.	Resource availability (−/+) Relative priority (+)

(+) positive influence, (−) negative influence, (−/+) both positive and negative influence

### Event 1: The formation of a new implementation network to explore the potential of Genie

The provider network and the commissioning network came together to create a new network that would explore the implementation of Genie. The formation of the new implementation network was influenced by two key factors; the political landscape, and the robust ‘evidence strength and quality’ of the intervention.

These two networks came together around a common interest, the implementation of Genie, and were enrolled in one network. The enrollment was encouraged and facilitated by the political landscape (relating to the CFIR concept ‘external policies’). This landscape influenced the commissioning network to design health and wellbeing services that would be ‘delivered as locally as possible and [be] person-centred’ (document: Health and Wellbeing Report); furthermore, the political landscape was a driving force promoting individual choice that would be achieved ‘through person-centred care planning and supported self-management of their health and wellbeing’ (document: a 5-year strategic plan). This political landscape encouraged the commissioning network to seek a service that would fulfil the need to achieve a person-centered care. Thus, the concept ‘external policies’ was influential in the early stages of this new network configuration.

The political landscape at the time meant the intervention was meaningful to the commissioning network as they were seeking a service that would fulfil the demands placed on them by the policy drive to deliver person-centred care. The intervention was considered meaningful to the commissioners because of the alignment between intervention focus and the political sphere.

People are desperately keen to do person centered planning, person centered care… in a way the Genie tool is the building block to allow some of this to happen.

(Interview, Commissioning-network)

The alignment between the commissioning network’s need and the intervention illuminates the importance of the CFIR concept ‘relative advantage’, that is, the intervention offering enough advantage to pursue implementation. This concept becomes a second influence in the early configuration of the implementation network.

A third influence in the configuration of the new implementation network was the ‘evidence strength and quality’ of Genie.

I think the evidence based is important. I think it’s been really useful to say

‘look at what [provider-network’s] work has demonstrated’

(Interview: Commissioning-network)

According to the commissioning network, the ‘evidence strength and quality’ behind Genie gave the intervention the meaning of being ‘the most informed way’ (observation: commissioning network) and a ‘valid approach’ (observation: commissioning network) for achieving their strategic objectives. Thus, the CFIR concept ‘evidence strength and quality’ was an influence making the intervention meaningful to the commissioning network and in doing so was an early influence in the configuration of the implementation network.

The creation of the implementation network was influenced by the alignment between the intervention and political landscape and supported by the robust ‘evidence strength and quality’ the intervention’s ‘relative advantage’ was validated. With these influences, the commissioning network financially invested in Genie and the two networks configured to jointly explore the implementation process.

### Event 2: Creating network stability by forming transactional associations

The implementation network was formalised when the commissioner network financially invested in Genie, and common interest and strategy were mutually agreed. At this juncture, the newly formed network’s stability increased, and the association between members became transactional in nature. This transactional association was apparent in how the network members gave meaning to their own and the other’s role in the network.

When you go and see a customer or a potential user group they expect when they license Genie, they can just use it without having to do anything

(Interview: Intervention-provider)

The intervention providers defined the commissioners as ‘the customers’ (interview), and in the context of the financial investment, the commissioners recognized they had ‘brought [Genie] for the city’ (observation: commissioning-network). Following the implementation network members taking on meaning in relation to each other, the transactional nature of the association between individuals from each ‘original’ network remained stable over an 18-month period.

Networks are fluid and their form is constantly (re)created, and the original implementation strategy was an influence in recreating the transactional association between members. Specifically, the way the members of the provider network agreed for the commissioning network to determine the details of the implementation strategy. Money and time were influential in encouraging the provider network to agree to this, and this relates to the CFIR concept ‘resource availability’. Based on previous research, the provider network considered the cost and work associated with implementation to be greater than they could afford. Therefore, they took the decision to withdraw from direct involvement in implementation. This illustrates the influence of the concept ‘resource availability’, and the way network components interact that can impact on network stability during the implementation process.

The original implementation strategy (see above) was determined by the commissioning network and was dependent upon ‘the navigation tender’ (observation: commissioning network). The strategy was to embed the intervention into roles within the community.

We’ve put the responsibility for training of people in the community, community groups, neighbour groups within the community navigation specification

(Interview: commissioning-network)

The proposed implementation strategy aligned to the commissioning network’s health and wellbeing strategy that outlined ‘community services will be the first port of call for people seeking help for themselves or others’ (document: Health and Wellbeing Report). With the community at the heart of the implementation strategy, the implementation process, and stability of the implementation-network, was contingent on an anticipated community landscape.

The stability of the implementation network was threatened by two influential threats. One was the failed tendering process that would have led to the community navigation roles being filled. This was the key element in the planned implementation strategy and was not successfully awarded. The second was the pressure of time, which was a negative influence affecting the stability of the network, as according to the provider network there was a pressing ‘need to put Genie on a footing where it is sustainable as an entity’ (interview: provider-network). With these two juxtaposing influences, the stability of the implementation network reduced, and the network began to evolve as implementation progress stalled.

### Event 3: Proving network resilience through a process of reflection and productive problematization

The original strategy employed by the implementation network (as defined by the commissioning network) proved to be unfeasible over time, which reduced the stability of the implementation network. In order to promote stability members of the provider network reflected on the process of implementation to date to understand the nature of the problems arising.

It is observed that the composition of networks becomes evident when things go wrong [29] and this was experienced here. Through this process, it became evident that there was little agreement on the pace of implementation between the two ‘original’ networks, which highlighted differences in expectations between network members and drove the evolution of the implementation network. The provider network reflected on working in the public sector as ‘moving slowly and sideward, compared to a commercial organization it is quite slow’ (interview: provider network). The latter was perceived by the commissioning network vis: ‘I think there’s some anxiety from the Genie-team about the speed that we’re going’ (observation). The implementation network remained relatively stable with an agreed focus that ‘Genie needs to work at scale’ (observation: commissioning network). This joint focus and commitment encouraged a period of reflection and evaluation of the implementation process to date in order to progress implementation and achieved ultimately through stabilizing the network.

With what the CFIR name ‘reflecting and evaluating’, the provider network considered the process and progress to date to explore alternative ways that could progress the implementation efforts further. Through the pursuit of the original implementation strategy, it became clear that there was limited ‘readiness for implementation’ in the community setting, which had not been foreseen due to a ‘gap between the commissioning-network and people on the ground’ (observation: provider network). Members of the provider network perceived a lack of promotional engagement of the intervention; this is significant because according to the CFIR, this is a key component of ‘readiness for implementation’.

Buy in is important, the people in the room had no buy in, they didn’t even know why they were there, they had just received an email [from the commissioning-network] to say they needed to attend.

(observation: provider network)

This highlights the importance of a network agreeing on the work necessary to reach the agreed focus and the division of labour of this, as failure to do so threatens implementation progress by destabilizing the network. However, through the process of reflecting and evaluating a need for a ‘Genie-engagement officer’ (observation: provider network) was proposed as a solution to progress implementation. The proposed officer would be responsible for promotional and pre-implementation activities. This process of reflection and evaluating illustrates the evolutionary nature of networks and the importance of an agreed focus and division of labour to maintain network stability, but above this also demonstrates that network resilience is a contributing factor towards implementation progress.

### Event 4: Network collaboration in creating the necessary facets for a context for implementation

Network resilience contributed towards the implementation network evolving from one of a transactional relationship between members, to a network with more collaborative relationships between members. This evolution emerged from the implementation not progressing as originally anticipated due to the conditions necessary for implementation being absent. Namely, the need for a Genie officer to promote the intervention. The implementation network was in agreement but what became evident was the various influences on different network members. As time has passed the concept ‘resource availability’ had become an influence preventing the commissioning network from fulfilling the role crucial for implementation ‘because there is just less money in the system’ (interview: commissioning-network). However, for members of the provider network with the lapse in time the CFIR concept ‘relative priority’ had become a greater influence than ‘resource availability’ once was. Specifically, as outlined above, there was a need for Genie to be a sustainable entity. Drawing upon their extended network (the research centre), the provider network was able to perform the organizational engagement work with the community that was deemed necessary to progress implementation (this was achieved through greater project management input). This illustrates the evolutionary nature of networks and how roles and the division of labour can be reconfigured to promote network stability and in doing so progress implementation.

The relationships between implementation network members transformed from a transactional customer-seller relationship to collaborators working jointly to achieve implementation. At this juncture, the implementation network was engaged in a process of enrolling new network members. Specifically, members of the community who would help to understand the localized implementation context and also deliver the intervention. The important role of the CFIR concept ‘evidence strength and quality’ in enrolling new members became clear. In the early stages of the configuration of the implementation-network, the ‘evidence strength and quality’ of the intervention had meaning for members of the commissioning network (those at the system level) when evidence was of health economics and trial-based data. However, when enrolling network members from the community (those at a local level), ‘evidence strength and quality’ was meaningful when it was of a case-study nature.

There were some familiar faces around the table as Genie was being presented. Many nodded with a smile of encouragement, but one (the lead nurse from the health centre we were sat in) looked less convinced, ‘that’s all very well but what I want to see is case studies, what has this done for people, I would like to see this’ she responded interrupting a moments pause.

(observation: provider-network)

The enrollment of members into an implementation network is, in part, contingent on the ‘evidence strength and quality’ behind the intervention, and this evidence demonstrating relevance to them (individually or organizationally), and this links to the CIFR concept ‘relative advantage’.

Securing enrollment of community organizations into the implementation network was achieved by demonstrating the ‘relative advantage’ of the intervention. However, the CFIR concept ‘resource availability’ was also influential in their enrollment into the implementation network. Specifically, there was concern over the capacity of community organizations to deliver the intervention.

Feedback has generally been positive, but some have stated that they do not have the time available to conduct a Genie intervention alongside their own work. [Branch of a national charity] suggested a referral system would be beneficial.

(observation: provider-network)

The intervention was perceived to have a ‘relative advantage’ to community organizations, but concerns regarding capacity were more influential and troublesome for the implementation of a facilitated intervention. The importance of a resilient and stable implementation network achieved by a commitment to the same focus is demonstrated here. With this commitment, the implementation network evolved once more to maintain stability. Implementation progressed when the provider network demonstrated flexibility in order to create the conditions required for implementation.

We need to tailor to each organization. They may want to be trained but they may want to be a referral pathway and we do Genie

(observation: provider-network)

The evolution of the implementation network to more collaborative relationships between members stabilized the network and allowed for the implementation process to continue. Enrolling new members into the network in order to make the context ready for implementation is a continuous process, and it holds the potential to transform the implementation network and context to a position where the necessary elements are in place to make it work and sustainable. At the time of writing, this identified potential to transform the implementation network has not materalised and thus is a predicted fifth stage in the implementaiotn process. However, what has been illustrated is the configuration and evolution of an implementation network that has demonstrated a resilient, flexible and collaborative approach that can strengthen a network to continue pursuit of implementation, and we hope to demonstrate the ability to also create a context ready for implementation.

## Discussion

The paper set out to explore the process of implementation in a community setting and give an account of the evolution of implementation processes. Through taking the network as the unit of analysis, the paper illustrates the influences that created and changed the phases of the implementation process. What has been demonstrated by this ethnographic approach is the importance of a flexible and resilient implementation network. This becomes all the more important when there is a misalignment between the imagined implementation context and the actual experienced context in which the necessary facets required for implementation do not exist.

The experience as documented by this study demonstrates how the political agenda influenced the configuration of the implementation network and the initial and continuing investment in the intervention, but that also that the political agenda (one of austerity) negatively impacted the process of implementation. This was most noticeable in terms of the effect the infrastructure required for implementation [19]. The process experienced here hints towards the local health and social care community operating at capacity with limited resource availability or flex to perform the work necessary for successful implementation as the required facets were created directly by the implementation network [15, 16].

Research of previous implementation strategies in different contexts suggests that organising detailed implementation strategies to achieve specific outcomes lacks adequate strategic development, which ultimately makes replication difficult [30]. This is also the case in relation to the strategy of building community connections and promoting patient involvement as part of implementation for SMS interventions. In terms of the intervention discussed here, the expected implementation outcomes for strategies included, establishing improved community connections and improved referrals to accessible patient resources [31]. What the study here shows is that in the instance of the original implementation strategy having to be abandoned, or the strategy not materialising as originally envisaged, making an intervention implementable emerges from network resilience and contingencies which arise as a result of attempts to make the intervention workable on the ground. These findings suggest that in addition to constructing and identifying implementation strategies in advance where rapid responses to implementation are necessitated by policy and health care providers, the response of networks provides a reverse understanding of what will work in a particular context to make an intervention workable. This may then become a prototype for looking at how an implementation strategy can be developed for second- and third-wave adopters of as in this case of SMS intervention.

We acknowledge that the current findings report on the implementation of a public health intervention within a UK community context and that they should be interpreted accordingly. However, with this note, there are lessons and implications to be taken forward by the networks involved, and there should also be considerations for any implementation processes taking place in a community setting.

### Implications

Through offering an account of the events in the implementation process, three lessons have been learnt.

The first implication for practice is understanding that enrolling organizations into the implementation network depends on the evidence strength and quality of evidence and that this is determined by the audience and stage of implementation. Most notably, an intervention is required to demonstrate value for money with health economic data to support the case for commissioning. Likewise, evidence of health improvement on a large population scale is valued by commissioners. In addition, interventions are also likely to require evidence of a case-study nature to engage specifically with organizations and the professionals working in these organizations where this is the case advice would be to select a case study example that aligns with the patient population and the organization support.

The second implication returns to the point made by Jefferies et al. [15, 16], which highlights the importance of communication between networks. It is the experience here that a significant element of the work was hidden to maintain relationships between members of the implementation network. This manifested in understanding the other, the other’s expectations of the relationship, the process and also the influences in respective settings. Based on this experience, there is a real need for early engagement work between network members to encourage early alignment on implementation strategies and division of labour.

The third implication is the need to concurrently engage on a system and organizational level. Influenced by a lack of capacity in the system, and the need to understand localized implementation contexts, there is a real need to engage early in pre-implementation work. This work is likely to be time-consuming and costly (in the human resources) and should include understanding the work patterns, culture, resource availability and flexibility of contexts. This paper has touched on the lack of capacity at a system level and organizational level. Troubleshooting to overcome these issues is key, and failure to engage on both levels is likely to lead to implementation delays and will almost certainly be a threat to sustainability.

### Conclusion

The CFIR was useful in illuminating influential actants creating the events in the implementation process. Firstly, at a commissioning level, the CFIR highlighted the need for intervention alignment with the contemporary political agenda, as well as providing sufficient relative advantage demonstrated through health economic evidence. Secondly, at a local level, the importance of resource availability (to ensure necessary capacity), there is a role for case-study evidence that is relatable to the ‘patient needs’ of the organization. Thirdly, on an individual level, there is a requirement for ‘reflection and evaluation’ to modify the implementation process as a result of learning. The concluding thoughts turn to consider the question of what is implementation success? Resilience and creativity of all involved in the process are required to engage with a process which is far more complex than the first imagined and in practice is dynamic and fraught with obstacles. However, if we move beyond the stringent dichotomy of implementation success and failure, with perseverance and resilience to respond accordingly, the process as outlined here, because implementation continues despite these obstacles, is, in its way, a success.

## Data Availability

The datasets during and/or analysed during the current study available from the corresponding author on reasonable request.

## Abbreviations

CCG: Clinical Commissioning Group
CFIR: Consolidated Framework for Implementation Research
CLAHRC: Collaboration for Leadership in Applied Health Research and Care
Genie: Generating Engagement in Network Involvement
NHS: National Health Service
NIHR: National Institute for Health Research
SMS: Self-management support
SOP: Standard operating procedure
